# The Impact of Expanded Testing for Multidrug Resistant Tuberculosis Using Geontype MTBDR*plus* in South Africa: An Observational Cohort Study

**DOI:** 10.1371/journal.pone.0049898

**Published:** 2012-11-30

**Authors:** Colleen F. Hanrahan, Susan E. Dorman, Linda Erasmus, Hendrik Koornhof, Gerrit Coetzee, Jonathan E. Golub

**Affiliations:** 1 Department of Epidemiology, Johns Hopkins Bloomberg School of Public Health, Baltimore, Maryland, United States of America; 2 Department of Epidemiology, UNC Gilling School of Global Public Health, Chapel Hill, North Carolina, United States of America; 3 Department of Medicine, Johns Hopkins School of Medicine, Baltimore, Maryland, United States of America; 4 National TB Reference Laboratory, National Health Laboratory Services, Johannesburg, South Africa; University of Cape Town, South Africa

## Abstract

**Introduction:**

Globally, multidrug resistant tuberculosis (MDR-TB) remains underdiagnosed. The Genotype MTBDR*plus®*, a rapid drug susceptibility testing (DST) assay used to detect resistance to isoniazid and rifampicin in the diagnosis of MDR-TB, has good diagnostic accuracy, but its impact on patient outcomes in routine practice is unproven. We assessed the clinical impact of routine DST using MTBDR*plus* in a single health district in South Africa.

**Methods:**

Data were collected on all adult pulmonary TB patients registered at 25 public health clinics in the periods before and after introduction of an expanded DST algorithm using MTBDR*plus* version 1.0.

**Results:**

We collected data on 1176 TB patients before implementation and 1177 patients afterwards. In the before period, measured MDR-TB prevalence among new cases was 0.7% (95% CI1.4–3.1%), and among retreatment cases 6.2% (95% CI:3.5–8.8%), *versus* 3.7% (95% CI:2.4–5.0, p<0.01) and 6.6% (95% CI:3.8–9.4%, p = 0.83) respectively after MTBDR*plus* introduction. The median times from sputum collection to MDR treatment in the before and after periods were 78 days (IQR:52–93) and 62 days (IQR:32–86, p = 0.05), respectively. Among MDR-TB cases, 27% (95%CI:10–44) in the before period converted sputum cultures to negative by 8 months following treatment initiation, while 52% (95%CI:38–66) converted in the intervention period (p = 0.04).

**Conclusions:**

The expanded use of MTBDR*plus* DST resulted in a substantial increase in the proportion of new cases identified as MDR-TB; though time to MDR treatment was reduced, it was still over two months. Culture conversion for MDR-TB patients improved after introduction of MTBDR*plus.* This work illustrates the mixture of successes and challenges resulting from increased access to rapid DST in a setting with a high TB burden.

## Introduction

Diagnosis of multidrug resistant tuberculosis (MDR-TB) represents a barrier to TB control, as only 7% of the estimated 440,000 emerging cases worldwide in 2008 were detected [1]. Global laboratory capacity for drug susceptibility testing (DST) to diagnose MDR-TB remains low–in 2010, just 13 of the 27 high MDR-TB burden countries reported at least 1 laboratory to perform DST per 5 million people [2]. Timely diagnosis of MDR-TB has the potential to improve treatment outcomes, reduce mortality, and reduce transmission [3].

Culture-based DST methods are hampered by slow turnaround times–culture on solid media can take 8–12 weeks for results, while a liquid culture system, the BACTEC MGIT 960 (BD, Franklin Lakes, NJ) has only reduced this timeframe to 3–4 weeks [4]. Line probe assays (LPA) are molecular methods for DST that can be performed directly on smear-positive sputum specimens or cultured isolates. One commercially available LPA, MTBDR*plus* version 1.0 (Hain LifeScience GmbH, Nehren, Germany), showed excellent sensitivity and specificity for rifampin resistance (98.1% and 98.7%, respectively) and lower accuracy for isoniazid resistance (84.3% sensitivity and 99.5% specificity) in a recent meta-analysis [5]. In a demonstration study set in a high-volume South African clinical laboratory, DST results were available within 1–2 days, with a total turnaround time of <7 days [6]. The MTBDR*plus* assay is technically complex and requires significant laboratory resources including specialized instrumentation, highly trained technicians, reliable electricity, and separate rooms for extraction, amplification, and hybridization. In 2008, the WHO endorsed the use of LPAs for DST as a rapid alternative to culture [7].

In 2009, South Africa introduced a new DST strategy, moving from using MGIT-based phenotypic DST performed only for TB patients with risk factors for MDR-TB, to using MTBDR*plus* on all bacteriologically confirmed TB patients. The goal of the present study was to evaluate the impact of this strategy change. We present data collected in the course of routine TB program operation in a single health district in South Africa from the periods before and after the introduction of the expanded DST algorithm using MTBDR*plus* version 1.0. We compared prevalence of drug resistance, uptake of DST, times to results and MDR treatment, and culture conversion and mortality among MDR patients before and after introduction of MTBDR*plus* in routine clinical services.

## Methods

### Study Design

**Figure 1:**
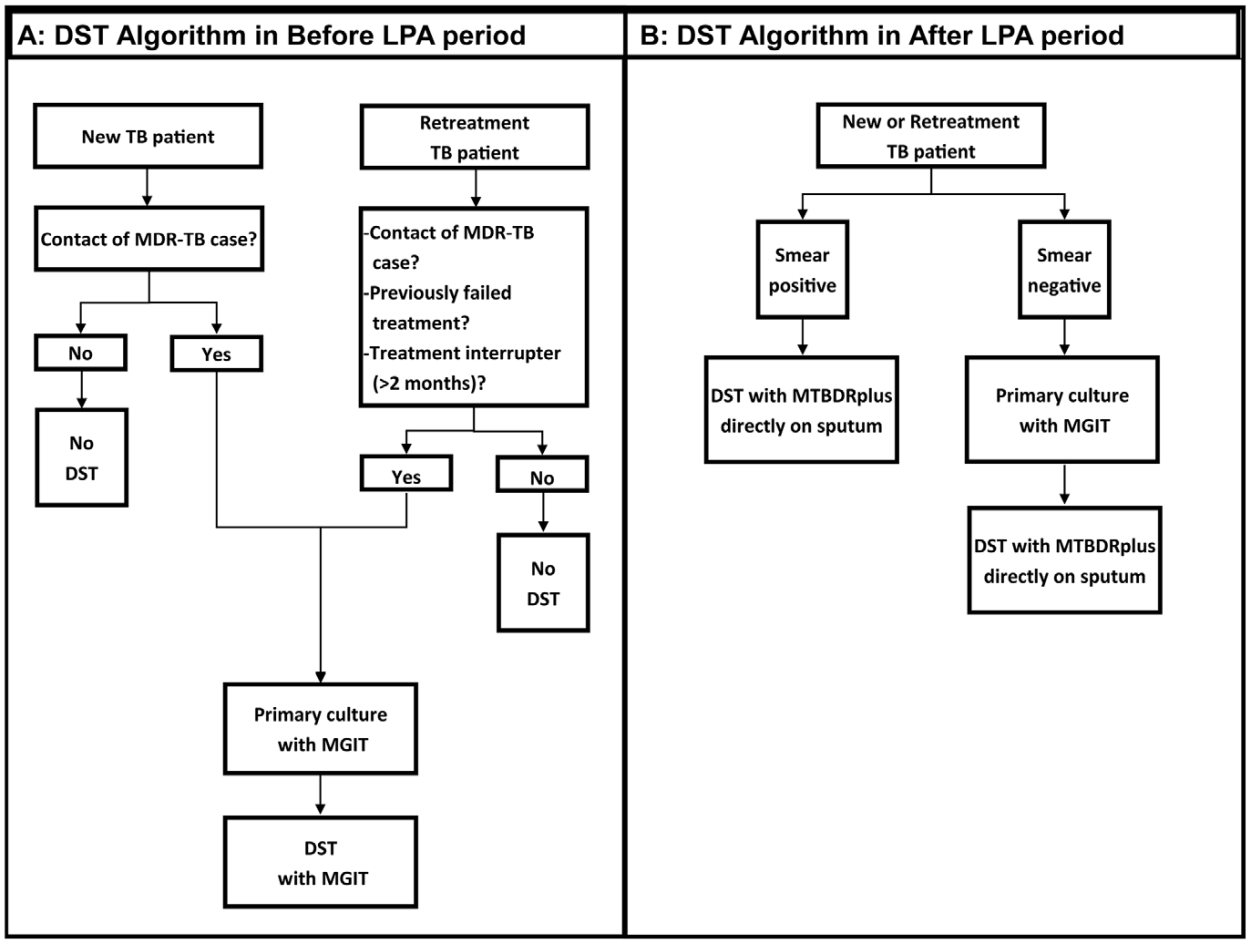
DST algorithm by study period. An illustration of the DST algorithm in the Before LPA period (A) and the After LPA period (B). Abbreviations: DST, drug susceptibility testing; LPA, line probe assay; TB, tuberculosis; MDR-TB, multidrug resistant tuberculosis.

We conducted a “before and after” cohort study in the Frances Baard health district in Northern Cape Province, South Africa. The expanded DST algorithm using MTBDR*plus* version 1.0, a commercially available LPA, was instituted in September 2009. Data on all newly registered adult (age ≥18 years) pulmonary TB patients from 25 public health facilities within the district were retrospectively collected through record reviews for two six-month time periods: prior to the introduction of the expanded algorithm from August 2007-January 2008 (the “Before LPA” period) and afterwards, from October 2009-March 2010 (the “After LPA” period). DST algorithms for both periods are presented in Figure 1. During the Before LPA period, DST was performed according to the 2004 National TB Control Guidelines [8] using MGIT for TB cases assessed by clinicians as being at high risk for MDR-TB. During the After LPA period, DST was performed using MTBDR*plus* on all TB cases with either a positive smear of grade ≥ scanty 8 (defined as at least 8 acid fast bacilli per 100 oil immersion fields [9]) or a positive MGIT culture.

### Laboratory Testing

Laboratory testing was performed under routine program conditions at the National Health Laboratory Services (NHLS) TB referral laboratory in Kimberley, South Africa. Sputa were collected according to TB program guidelines [8], [10] and couriered daily to the laboratory. Specimens were concentrated via centrifugation and decontaminated using N-acetyl L-cysteine-NaOH, before resuspension in phosphate buffer (pH 6.8). Fixed smears were stained with auramine then examined with florescence microscopy. Slides were graded according to the International Union Against Tuberculosis and Lung Disease scale [9]. MGIT tubes were inoculated using 0.5 mL of the digested, resuspended sputum, and were incubated and read daily per manufacturer instructions [11]. Cultures were considered negative after 35 days without growth. Positive MGIT cultures were examined for acid fast bacilli by Ziehl-Neelson staining.

During the Before LPA period, DST was performed upon clinician request and according to manufacturer protocol on positive MGIT cultures using the BD BACTEC SIRE Drug Kit [12]. During the After LPA period, DST was performed using MTBDR*plus* version 1.0 directly on sputum samples graded ≥scanty 8, and on MGIT cultures from sputum samples graded<scanty 8 or negative. Briefly, DNA was extracted from 1 mL of positive MGIT culture, or 0.5 mL of concentrated resuspended sputum sample according to manufacturer’s protocol [13]. Hybridization was done using the Hain Lifescience GT Blot 48. For MTBDR*plus* testing, resistance to isoniazid (INH) and rifampin (RIF) were interpreted for samples positive for *M. tuberculosis* complex. If resistance was indeterminate by MTBDR*plus*, that test was repeated using the MGIT culture isolate, if available. Strips with positive control bands and no band for *M. tuberculosis* complex were reported as non-tuberculous Mycobacteria, and no DST results were read or reported. Culture-based DST was not performed in order to confirm MTBDRplus findings. Laboratory test data were captured routinely using the NHLS electronic data management system. Patient data abstracted from clinic records were matched to laboratory data through laboratory identification numbers and unique patient identifiers.

### Statistical Analysis

Statistical analyses were conducted using Stata 11.2 (StataCorp LP, College Station, TX). Standard descriptive statistics were used to characterize the study population at TB diagnosis. Pearson’s chi-square was used to compare categorical variables and the Wilcoxon rank-sum test was used for continuous variables. Kaplan-Meier curves were used to estimate time to MDR treatment for MDR-TB patients. As the time to treatment during the Before LPA period was likely an underestimate due to unobserved times for undetected MDR cases, a simple model was created to estimate the true time to appropriate treatment during this period. In this model, during the Before LPA period, individuals who did not have the outcome (MDR treatment) were added to the population of MDR-TB cases and were censored after six months spent on incorrect treatment. Estimates are presented for data as collected as well as modeled data for this study period.

Outcomes for MDR-TB patients were administratively censored at eight months following registration in order to ensure comparable follow-up between the study periods. Culture conversion among MDR patients was considered to be two consecutive negative cultures, at least one month apart. Mortality among MDR patients was estimated using Kaplan-Meier curves and compared using the log-rank statistic.

### Ethics Approval

This study was approved by the Johns Hopkins Medicine (NA_00031006) and University of the Witwatersrand (M090679) institutional review boards, and had the approval of the Department of Health of the Northern Cape Province, Kimberley, South Africa. The need for written informed consent was waived by both review boards as participant information was gathered solely through the review of existing medical records collected during the course of routine patient care, the study carried minimal risk to the participants, the data were analyzed anonymously, and the study was conducted with a government entity as operational research in order to evaluate an ongoing public health program.

## Results

### Characteristics of the Study Population at TB Diagnosis

Overall, 3734 TB patients were recorded in the TB registries of the 25 public health facilities–1826 (49%) in the Before LPA period and 1908 (51%) in the After LPA period. During the Before LPA period, 237 extrapulmonary (13%) and 413 pediatric cases (22%) were excluded, while during the After LPA period, 229 extrapulmonary (12%) and 502 pediatric cases (26%) were excluded. Characteristics of the remaining 2353 adult pulmonary TB patients are presented in Table 1.

**Table 1:** Characteristics of all TB patients and MDR-TB patients at the time of TB diagnosis.

	All TB Patients			MDR-TB Patients		
Characteristic	Before LPA	After LPA	p-value*	Before LPA	After LPA	p-value*
**n (%)**	1176 (50%)	1177 (50%)	–	26 (33%)	52 (67%)	<0.001
**Males, n (%)**	649 (55%)	644 (55%)	0.849	12 (46%)	20 (38%)	0.515
**Median age (IQR)**	36 (29–45)	38 (30–46)	0.054	36 (31–37)	36 (27–41)	0.162
**Unemployed, n (%)**	999 (86%)	986 (85%)	0.496	23 (88%)	44 (85%)	0.399
**Patient status at** **registration, n (%)**						
**New**	852 (72%)	872 (74%)	0.369	6 (23%)	32 (62%)	0.001
**Retreatment**	324 (28%)	305 (26%)		20 (77%)	20 (38%)	
**Smear microscopy status at** **registration, n (%)**			<0.001			0.059
**Positive**	767 (65%)	664 (56%)		20 (77%)	30 (58%)	
**Negative**	275 (23%)	493 (42%)		5 (19%)	22 (42%)	
**Unknown/not done**	134 (11%)	20 (2%)		1 (4%)	0 (0)%)	
**HIV Status, n (%)**			<0.001			0.598
**Positive**	667 (57%)	685 (58%)		15 (58%)	36 (69%)	
**Negative**	299 (25%)	413 (35%)		6 (23%)	9 (17%)	
**Unknown**	210 (18%)	79 (7%)		3 (19%)	7 (13%)	
**Median CD4 count among HIV-infected individuals at TB diagnosis (IQR, cells/µl)** **	185 (82–336)	176 (77–322)	0.773	297 (170–399)	174 (44–277)	0.082
**On ART at TB diagnosis, n (% among HIV+)**	101 (18%)	188 (29%)	<0.001	4 (27%)	23 (64%)	0.018
**On cotrimoxazole at TB diagnosis,** **n (% among HIV+)**	456 (87%)	558 (84%)	0.320	24 (92%)	52 (88%)	0.599

Abbreviations: LPA, MTBDR*plus* line probe assay, IQR, interquartile range; HIV, human immunodeficiency virus; ART, antiretroviral therapy; TB, tuberculosis.

* p values are for comparison of the Before LPA period versus the After LPA period.

** CD4 cell counts within 6 months of TB diagnosis were recorded for 502 (75%) during the Before LPA period and 612 (89%) during the After LPA period.

HIV testing of TB patients increased between study periods, with 82% of patients in the Before LPA period having known HIV status compared to 93% in the After LPA period (p<0.001). The proportion of TB patients on antiretroviral therapy (ART) at TB diagnosis increased from 18% (95% CI: 15–21) during the Before LPA period to 29% (95% CI: 26–32) during the After LPA period (p<0.001).

During the Before LPA period, culture was performed on 67% of TB cases as part of the initial TB diagnostic workup, including 82% of smear negatives and 62% of smear positives. During the After LPA period, use of culture increased to 80% of TB cases (p<0.001), including 86% of smear negatives and 77% of smear positives.

### DST and MDR-TB during the Two Study Periods

**Figure 2:**
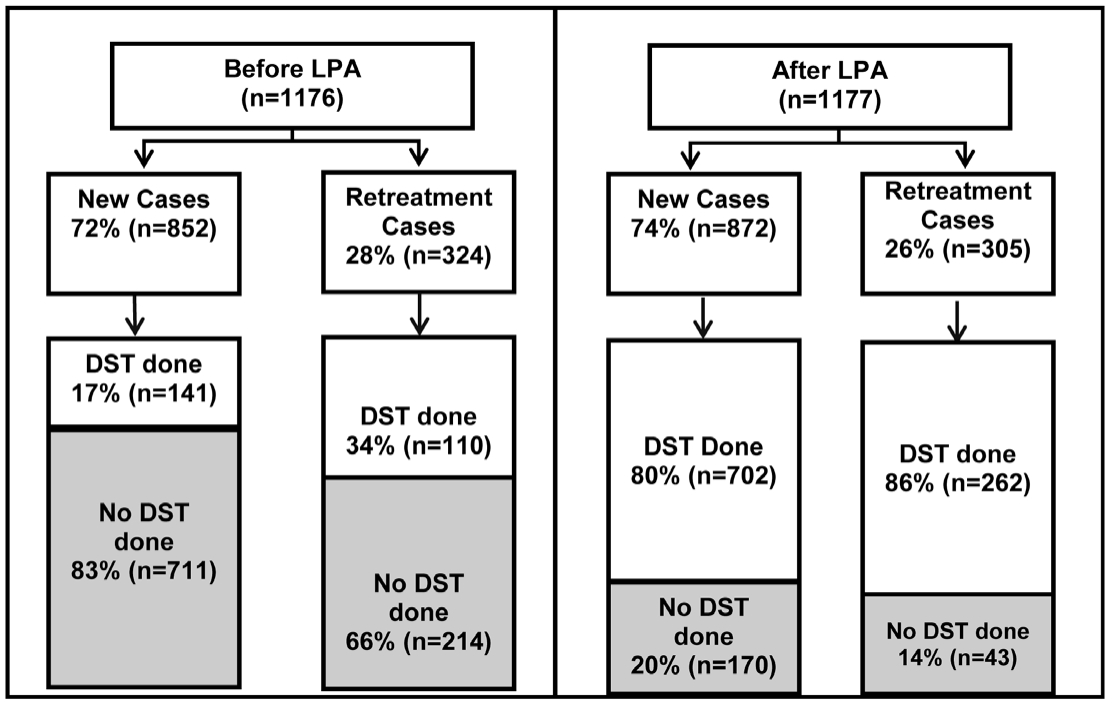
DST by patient category and study period. A breakdown of whether DST was performed or not, by study period (Before and After LPA) and patient category (New versus Retreatment). Abbreviations: LPA, MTBDR*plus* line probe assay; DST, drug susceptibility testing.

After introduction of the expanded testing algorithm, DST was more widely applied (Figure 2). During the Before LPA period, 251/1176 (21%, 95% CI: 19–23) TB cases had DST performed, compared to 962/1177 (82%, 95% CI: 80–84) cases in the After LPA period (p<0.001). Table 2 presents drug resistance by study period. In the Before LPA period, 26 cases of MDR-TB were identified among 1176 TB patients (2.2%, 95% CI: 1.4–3.1%). In the After LPA, twice as many (52) cases were found among 1177 TB patients, for a prevalence of 4.5% (95%CI: 3.2–5.6%, p 0.003). The increase in diagnosed MDR-TB cases was mainly among new cases–during the After LPA period 3.7% (95%CI: 2.4–5.0) of new cases had MDR-TB diagnosed, while during the Before LPA period, only 0.7% (0.01–1.2%) of new cases had MDR-TB detected (p<0.001). We also observed an increase in RIF and INH mono-resistance between periods. During the Before LPA period, 2.4% (95%CI:1.5–3.3) of all cases were diagnosed with INH mono-resistance, while in the After LPA period, 4.2% (95%CI:3.0–5.3) were diagnosed (p = 0.015). During the Before LPA period, 0.2% (95%CI:0–0.4) were diagnosed with RIF mono-resistance, while 1.8% (95%CI:1.0–2.5) were diagnosed during the After LPA period (p<0.001).

**Table 2:** Drug resistance by study period.

	Before LPA (n = 1176)		After LPA (n = 1177)		p-value
	n	% (95% CI)	n	% (95% CI)	
**MDR, all**	26	2.2 (1.4–3.1)	52	4.4 (3.2–5.6)	0.003
**New cases**	6	0.7 (0.01–1.2)	32	3.7 (2.4–5.0)	<0.001
**Retreatment cases**	20	6.2 (3.6–8.8)	20	6.6 (3.8–9.4)	0.838
**INH mono-resistance, all**	28	2.4 (1.5–3.3)	49	4.2 (3.0–5.3)	0.015
**New cases**	9	1.1 (0.4–1.7)	35	4.0 (2.7–5.3)	<0.001
**Retreatment cases**	19	5.9 (3.3–8.4)	14	4.6 (2.2–6.9)	0.475
**RIF mono-resistance, all**	2	0.2 (0–0.4)	21	1.8 (1.0–2.5)	<0.001
**New cases**	0	0	13	1.5 (0.6–2.3)	<0.001
**Retreatment cases**	2	0.6 (0–1.5)	8	2.6 (0.8–4.4)	0.045
**Any resistance** * **, all**	56	4.8 (3.5–6.0)	121	10.3 (8.5–12.0)	<0.001
**New cases**	15	1.8 (0.8–2.6)	80	9.2 (7.2–11.1)	<0.001
**Retreatment cases**	41	12.7 (9.0–16.3)	41	13.4 (9.6–17.3)	0.767
**MDR yield (among those tested)**		10.4 (6.9–14.8)		5.2 (3.8–6.6)	<0.001
**Any resistance yield (among those tested)**		20.3 (15.3–25.3)		12.2 (10.2–14.3)	0.001

Abbreviations: LPA, Hain MTBDR*plus* line probe assay; CI, confidence interval; MDR, multi-drug resistant; INH, isoniazid; RIF, rifampin.

* Any resistance refers to resistance to either INH or RIF, or both.

### Test Turnaround Time and Time to MDR Treatment

**Figure 3:**
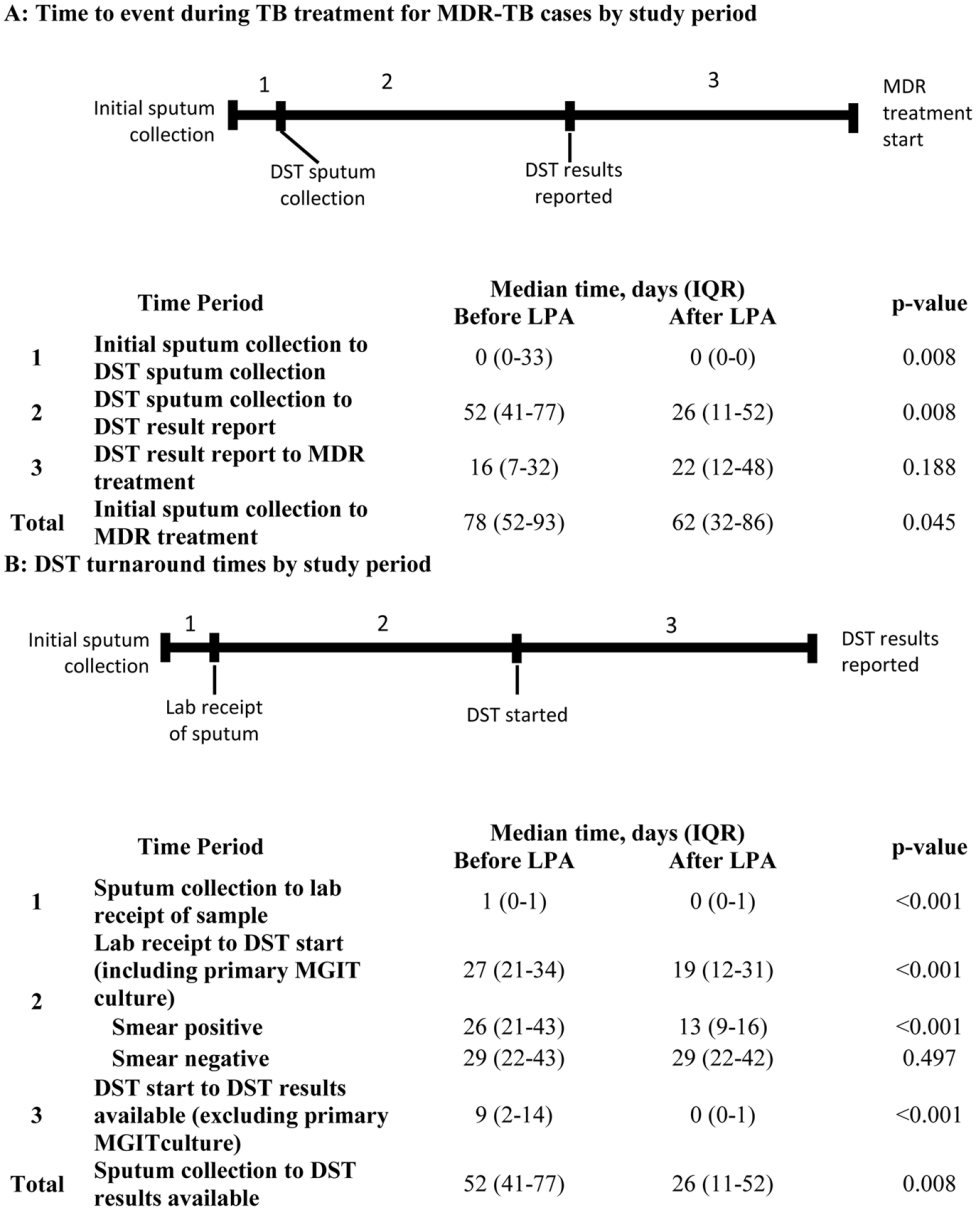
Time to treatment and drug susceptibility test turnaround times by study period. An illustration of critical events in the time to MDR treatment and DST turnaround. Abbreviations: DST, drug susceptibility testing; LPA, MTBDR*plus* line probe assay; MDR, multi-drug resistant tuberculosis; IQR, inter-quartile range.

Figure 3A presents a decomposition of the time to MDR treatment. The median time from sputum collection to DST results (Figure 3A, time period 2) during the After LPA period was half that observed in the Before LPA period (Before LPA, 52 days [IQR: 41–77]; After LPA, 26 days [IQR: 11–52 days]; p 0.008).

The median time from initial sputum collection to start of MDR treatment during the Before LPA period was 78 days (IQR: 52–93), and was significantly shorter during the After LPA period at 62 days (IQR: 32–86, p 0.045). For smear positive cases during the After LPA period, the median time to MDR treatment was 54 days (IQR: 31–66), while during the Before LPA period, the median time to treatment was 79 days (IQR: 51–95, p 0.272). For smear negative cases during the After LPA period, time to treatment was 73.5 days (IQR: 43–94) compared to 89 days (IQR: 80–104, p 0.279) during the Before LPA period. The median time from available DST result to appropriate MDR treatment start (Figure 3A, time period 3) was 6 days longer during After LPA period (22 days, [IQR: 12–48]) as compared to the Before LPA period (16 days, [IQR: 7–32]), but the difference was not statistically significant (p 0.188).

Laboratory turnaround times are presented in Figure 3B. The median time from lab receipt of sputum to the start of DST, which included any necessary primary MGIT culture, was longer in the Before LPA period (27 days, [IQR: 21–34] compared to the After LPA period (19 days, [IQR: 12–31], p<0.001). Smear positive sputum samples during the After LPA period, which should not require primary MGIT culture, had a median delay of 13 days to start DST (IQR:9–16 days). Once DST was initiated, however, results were available a median of 9 days earlier during the After LPA period as compared to Before (p<0.001), with most results available within one day during the After LPA period (median time to results 0 days, IQR: 0–1).

**Figure 4:**
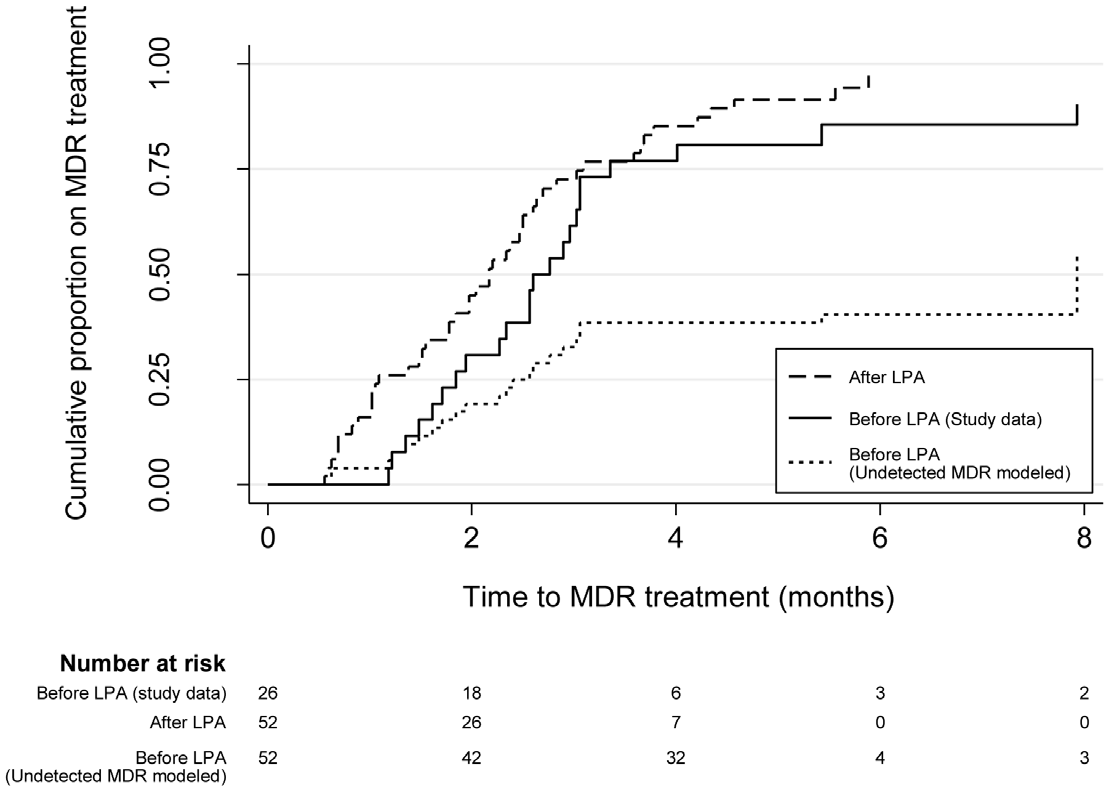
Time to MDR treatment among MDR-TB patients, by study period. Kaplan-Meir curves for time from initial sputum collection to MDR treatment by study period, including a modeled Before LPA period where the number of MDR-TB cases during the Before LPA period was augmented to match that observed during the After LPA period. Abbreviations: LPA, MTBDR*plus* line probe assay; MDR, multidrug resistant tuberculosis.

Figure 4 shows the Kaplan-Meir curves for time to MDR treatment by study period. Based on the observed MDR-TB prevalence in the After LPA period, the number of MDR-TB cases observed in the Before LPA period was likely an underestimate. For exploratory purposes, we augmented the number of MDR-TB cases during the Before LPA period to match that observed during the After LPA period, by including 26 undetected MDR cases in the Before LPA period. In this modeled Before LPA population of MDR-TB cases, only 44% were placed on appropriate treatment.

### MDR Patient Outcomes

The majority (64%) of MDR-TB patients during the Before LPA period were on an MDR treatment regimen of pyrazinamide, ethambutol, amikacin, ciprofloxacin and ethionimide, while during the After LPA period, the majority (66%) were on a regimen of pyrazinamide, kanamycin, ofloxacin, ethionimide and terizidone.

Among treated MDR-TB patients in the After LPA period, conversion of sputum cultures to negative occurred within 8 months of the initial clinic visit for 52% (95%CI:38–66), and the median time to culture conversion was 5.7 months (IQR:4.6–7.3). Median time to culture conversion for those in the Before LPA period could not be calculated as only 27% (95%CI:10–44, p 0.036) converted their cultures by 8 months. By smear status, more smear positive MDR cases in the After LPA period culture converted (62% [95%CI: 42–79]) compared to during the Before LPA period (22% [95%CI 6–48], p 0.008), while conversion among smear negative cases differed between the two periods, but the difference was not statistically significant (Before LPA: 80% [95%CI: 28–99]; After LPA: 53% [95%CI:28–77], p 0.279).

**Figure 5:**
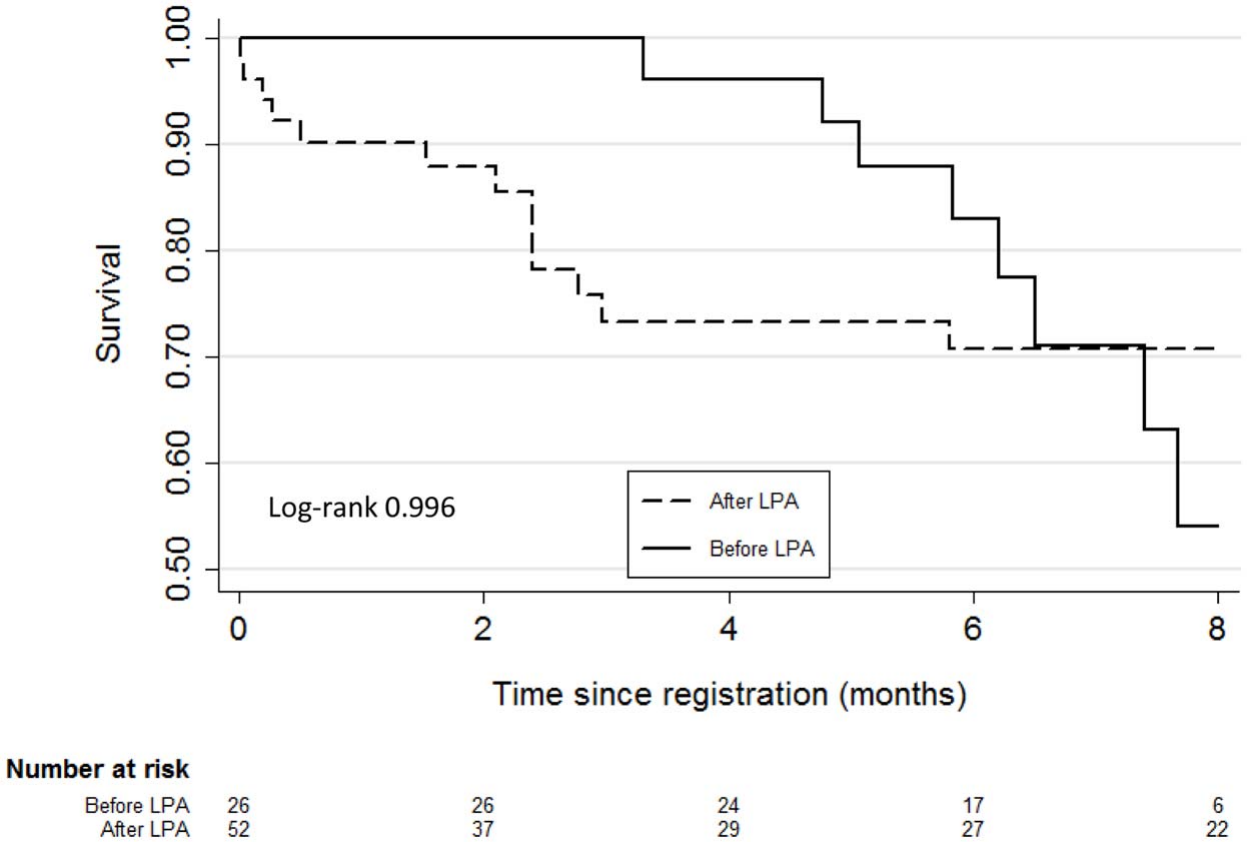
All-cause mortality by 8 months following initial registration among all MDR patients, by study period. Kaplan-Meir curves for time from initial TB case registration to death by all causes among all MDR patients, by study period**.** Abbreviation: LPA, MTBDR*plus* line probe assay.

During the Before LPA period eight deaths occurred by eight months following the initial TB diagnosis visit (57/100 PY, 95%CI:29–114/100 PY), while there were 13 deaths during the After LPA period (60/100 PY, 95%CI:35–104/100 PY). The incidence rate ratio for mortality comparing the After LPA period to the Before LPA period was 1.06 (95%CI:0.41–2.94, p 0.458). The timing of deaths among MDR patients was different by study period (Figure 5). Deaths during the After LPA period tended to be earlier– six (46%) of the deaths occurred within two months of registration, whereas in the Before LPA period there were no deaths within two months of registration. The majority of deaths during both study periods occurred among HIV-infected individuals (Before LPA: 75%, (95%CI:45–100); After LPA: 77%, (95%CI:54–100), p 0.917).

## Discussion

In this setting, one with a high burden of both TB and HIV, implementation of the expanded DST algorithm using MTBDR*plus* improved several important outcomes. Twice the number and proportion of MDR-TB cases was detected after introduction of the expanded testing algorithm compared to before, when DST was targeted to those thought to be at high risk for drug resistance. Similar increases in case finding of mono-resistance were also observed. Implementation of the expanded algorithm with MTBDRplus resulted in a dramatic increase in the application of DST among new TB cases and a substantial increase in the number and proportion of new cases identified as MDR or mono-resistant. Focusing DST on retreatment patients has been justified by the argument that those without a history of prior TB treatment are less likely to have resistance [14]. We confirmed that the proportion of retreatment cases having MDR-TB was higher than the proportion of new cases with MDR-TB even after implementation of expanded DST. Importantly, however, after implementation of expanded DST almost two-thirds of total MDR-TB cases were new TB cases, demonstrating the limitations of a DST testing strategy targeted to retreatment TB cases. Since new patients with MDR-TB most likely reflect acquisition through transmission, rather than resistance acquired through inappropriate treatment, our results underline the importance of control measures for prevention of MDR-TB transmission. Rapid identification of mono-resistant TB cases is also important in order to prevent serial acquisition of resistance, and development of acquired MDR-TB.

MDR-TB patients in the After LPA period were almost twice as likely to experience conversion of sputum cultures from positive to negative by eight months following treatment initiation. Although this beneficial effect may be partially due to a change in MDR treatment regimen and an increase in ART coverage between the two time periods, it nevertheless illustrates the value of identifying MDR-TB cases through expanded testing so that more individuals can be placed on appropriate treatment and transmission can be interrupted.

Our results identified several processes that require strengthening. Using MTBDR*plus* rather than MGIT for DST resulted in a shorter time from sputum collection to start of MDR treatment. However, even among smear positive individuals (in whom MTBDR*plus* can be performed directly on sputum), appropriate MDR-TB therapy was initiated a median of two months after initial sputum collection. Factors at the patient, clinic and laboratory level can contribute to this delay in treatment. In this study, the time from laboratory receipt of sputum to initiation of DST using MTBDR*plus* for smear positive specimens was almost two weeks, although we could not ascertain whether this delay was due to the need to repeat DST on the primary MGIT isolate or other factors. Whether performed directly on sputum or on positive MGIT culture, laboratory testing by MTBDR*plus* proved to be rapid–results were available the same day that testing was initiated. If MTBDR*plus* were introduced in a context where culture was not possible, we estimate that a significant proportion (42%) of MDR-TB cases would be missed by performing the test only on smear positive TB cases.

Troublingly, we found that after the introduction of the expanded testing algorithm using MTBDR*plus,* it took longer to initiate MDR treatment once DST results were known. During both time periods, MDR-TB treatment was provided only at a 30-bed inpatient unit that was often operating at capacity. Without provisions for additional inpatient treatment capacity or alternatives such as community-based MDR-TB treatment, this bottleneck will blunt any potential gains of universal access to DST in South Africa and elsewhere.

The before/after study design is an important limitation of this study, as it is subject to bias by temporal trends. It is possible that the higher prevalence of MDR-TB reported in the After LPA period was a result of a true increase in disease occurring over time in this community, rather than a reflection of increased detection due to expanded DST. However, it is unlikely that a doubling in MDR-TB prevalence occurred in the 18 months between study periods. Further, the observed increase in MDR-TB prevalence only among new TB cases and not among retreatment cases lends credence to the conclusion that the rise in MDR-TB reflected increased detection. Additional changes between the study periods including increased uptake of HIV testing, an increase in ART coverage and an improvement in MDR-TB treatment regimen likely impacted MDR patient outcomes such as culture conversion and mortality. Comparisons of these outcomes between study periods should be made cautiously, as the number of MDR-TB patients from this single health district does not allow for meaningful adjustment to control for potential confounding factors. Although the prevalence of MDR-TB is alarmingly high in South Africa and other countries, it still remains a relatively rare form of TB, and future studies examining the impact of MDR diagnostics should include sufficient patients to examine these important confounders.

This was an operational cohort study set within the context of regular TB control program activities, using routinely collected clinic and laboratory data. Diagnostic tests were conducted as requested by clinicians and therefore may not have exactly followed the recommended algorithm. These real world conditions are ideal for illustrating the impact that this diagnostic test and strategy can have in the very setting where implementation is intended. Further, an operational study can identify problem areas, such as the bottleneck in transitioning newly identified MDR-TB patients into appropriate treatment, which if rectified might lead to better patient outcomes.

Understanding the impact of MTBDR*plus* and the expanded DST algorithm in this study depends on comparing patient outcomes between the two study periods. As we illustrated, the time to appropriate treatment observed for MDR-TB patients in the Before LPA period is likely an underestimate, as perhaps half of all MDR-TB cases during this period were missed, and therefore time to MDR treatment was not observed. Likewise, estimates of the impact of testing on mortality among MDR-TB are subject to bias, as MDR-TB cases during the After LPA period were more likely to be detected earlier, and thus mortality occurring shortly after diagnosis would be correctly classified as MDR-TB mortality rather than misclassified as non-MDR-TB mortality as in the Before LPA period. These data represent a conservative underestimate of the effect of expanded testing for MDR-TB using the MTBDR*plus.*

The manufacturer has recently released the MTBDR*plus* version 2.0, an improved version of the test which has shown good accuracy in a validation study when used directly on both smear negative and positive sputum samples [15]. This potentially eliminates the need to first culture smear negative sputum samples, which should translate to comparable turnaround times for both smear negative and positive samples. Our data suggest that the impact of MTBDR*plus* version 1.0 was greater for smear positive patients compared to smear negatives (reduced time to treatment and increased culture conversion). The benefit to patients using this newer version may exceed what is demonstrated here, however further studies of version 2.0 in routine clinical practice are needed.

Since this study was conducted, South Africa has revised their TB diagnostic algorithm and is rolling out Xpert MTB/RIF (Cepheid, Sunnyvale, CA) as the initial diagnostic in all those suspected of TB [16]. Xpert MTB/RIF is a molecular assay endorsed by the WHO [17] with good accuracy for TB identification, rapid time to results (∼ two hours) and the ability to simultaneously detect resistance to rifampicin as an indicator of MDR-TB [18]. Xpert offers advantages over MTBDR*plus*: it can be performed directly on both smear positive and negative sputum, can be used to diagnose TB and rifampicin resistance, and is technically much simpler, using a fully integrated sample processing instrument [19]. Although the Xpert-based South African algorithm maintains expanded access to DST, Xpert is implemented at the point of the centralized laboratory, as with MTBDR*plus*, which may blunt some of the assay’s potential impact [20] and result in continued delays in time to results. Additionally, Xpert does not test for isoniazid resistance, thus this algorithm is likely to miss those with isoniazid mono-resistance. Finally, without meaningful expansion of access to MDR-TB treatment, Xpert will have no impact on decreasing the treatment bottleneck observed here.

Overall, our study illustrates the mixture of successes and challenges resulting from increased access to DST, and provides important information to guide program strengthening. Compared with the Before LPA strategy, under the expanded testing strategy more MDR-TB cases and mono-resistant were identified, the total time from initial sputum collection to initiation of appropriate MDR-TB treatment was shorter, and a higher proportion of MDR-TB patients experienced conversion of sputum cultures to negative within eight months of treatment initiation. However, time-to-appropriate MDR-TB treatment in this setting was still measured in months rather than days. A rapid, simple test that allows for decentralized testing has the potential to meaningfully decrease the time to DST results [18]. As shown in this study, an already overburdened public health system may not be able to keep pace with an increase in MDR-TB resulting from increased drug susceptibility testing, and expansion of MDR-TB treatment facilities and strategies will be required. As more new TB diagnostics are put into routine program use, assessments of impact on clinical outcomes and the health system will be critical. Diagnostic accuracy in the laboratory is arguably only a surrogate marker for outcomes of real importance – reduction of morbidity and mortality.
