# Correction: The Impact of Expanded Testing for Multidrug Resistant Tuberculosis Using Geontype MTBDR*plus* in South Africa: An Observational Cohort Study

**DOI:** 10.1371/annotation/47420ec0-db00-4994-a0de-86b5e5d713f4

**Published:** 2013-06-25

**Authors:** Colleen F. Hanrahan, Susan E. Dorman, Linda Erasmus, Hendrik Koornhof, Gerrit Coetzee, Jonathan E. Golub

Author Johnathan E. Golub is incorrectly associated with the following affiliation: 

2 Department of Epidemiology, UNC Gilling School of Global Public Health, Chapel Hill, North Carolina, United States of America.

The correct affiliations for Johnathan E. Golub are the following:

1 Department of Epidemiology, Johns Hopkins Bloomberg School of Public Health, Baltimore, Maryland, United States of America

3 Department of Medicine, Johns Hopkins School of Medicine, Baltimore,

Maryland, United States of America

